# Chromosome-Scale Genome Assembly of the Freshwater Snail *Semisulcospira habei* from the Lake Biwa Drainage System

**DOI:** 10.1093/gbe/evad208

**Published:** 2023-11-28

**Authors:** Osamu Miura, Atsushi Toyoda, Tetsuya Sakurai

**Affiliations:** Faculty of Agriculture and Marine Science, Kochi University, Nankoku, Kochi, Japan; Department of Genomics and Evolutionary Biology, National Institute of Genetics, Mishima, Shizuoka, Japan; Faculty of Agriculture and Marine Science, Kochi University, Nankoku, Kochi, Japan

**Keywords:** *Semisulcospira*, Cerithioidea, chromosome-scale assembly, B chromosome

## Abstract

*Semisulcospira habei* is a freshwater snail species endemic to the Lake Biwa drainage and belongs to a species group radiated within the lake system. We report the chromosome-scale genome assembly of *S. habei*, including eight megascaffolds larger than 150 Mb. The genome assembly size is about 2.0 Gb with an N50 of 237 Mb. There are 41,547 protein-coding genes modeled by ab initio gene prediction based on the transcriptome data set, and the BUSCO completeness of the annotated genes was 92.2%. The repeat elements comprise approximately 76% of the genome assembly. The Hi-C contact map showed seven well-resolved scaffolds that correspond to the basic haploid chromosome number of *S. habei* inferred from the preceding karyotypic study, while it also exhibited one scaffold with a complicated mosaic pattern that is likely to represent the complex of multiple supernumerary chromosomes. The genome assembly reported here represents a high-quality genome resource in disentangling the genomic background of the adaptive radiation of *Semisulcospira* and also facilitates evolutionary studies in the superfamily Cerithioidea.

**Significance**

There are fascinating examples of adaptive radiation in the ancient lakes. The Japanese ancient lake, Lake Biwa, harbors many endemic species, including a species flock of the gastropod genus *Semisulcospira*. The *Semisulcospira* snails in Lake Biwa can be a useful model system to study the mechanisms of species diversification, yet no high-quality genomic reference was available in this group. We report a chromosome-scale genome assembly of *Semisulcospira habei*, which will serve as an essential genomic resource for future research on the adaptive radiation of *Semisulcospira* and, more broadly, for evolutionary studies of the superfamily Cerithioidea.

## Introduction

Lake Biwa is an ancient lake in central Japan with approximately 4 million years of history and harbors more than 60 endemic species and subspecies (Nishino 2012). The freshwater snails in the genus *Semisulcospira* in the Lake Biwa drainage system involve 18 species (Sawada and Fuke 2022, 2023), exhibiting the highest within-genus diversity among all Lake Biwa endemic taxa. Two genetically and morphologically distinct *Semisulcospira* groups exist in Lake Biwa: the *Semisulcospira niponica* group and the *Semisulcospira nakasekoae* group (Sawada and Fuke 2022). Each group involves nine species. Recent genome DNA studies based on the reduced representation sequencing technique demonstrated that these endemic *Semisulcospira* groups concurrently diversified in response to the enlargement of the lake about 0.4 million years ago (Miura et al. 2019). Their rapid species diversification within the limited geographical scale provides an ideal study system to evaluate the genetic background of species diversification, as shown in cichlid fishes in ancient African lakes (Malinsky et al. 2018; Nakamura et al. 2021) and finches in the Galapagos Islands (Lamichhaney et al. 2015, 2018).

**Fig. 1.**
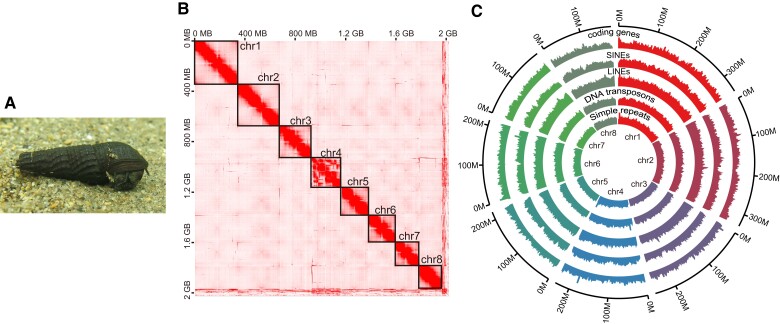
(*A*) The picture of *S. habei* at Uji River. (*B*) Hi-C contact map showing eight megascaffolds. (*C*) The Circos plot exhibits the distribution of coding genes, SINEs, LINEs, DNA transposons, and simple repeats in eight megascaffolds. The numbers of genes and repeat elements were counted by a sliding window of 1 Mb moving a step of 0.5 Mb. The scale of the maximum value of the *y* axis was set to 60 for coding genes, while it was set to 650 for the other repeat elements.

*Semisulcospira habei* (fig. 1*A*) belongs to the *S. niponica* group and is the only living species that also appears in the fossil records from the Paleo-Lake Biwa deposits (Matsuoka 1987; Nishino and Watanabe 2000). Other living *Semisulcospira* species in Lake Biwa were absent from the fossil records, perhaps because of their recent diversification (Miura et al. 2019). This fossil evidence suggests that *S. habei* is a candidate stem species that had seeded the radiation of the *S. niponica* group. The high-quality draft genome assembly of *S. habei* should be an essential resource to disentangle ecological and evolutionary mechanisms of species radiation in the *Semisulcospira* snails, including the identification of genes that facilitate the rapid species diversification of the *Semisulcospira* snails in Lake Biwa.

## Results and Discussion

The superfamily Cerithioidea includes more than 200 genera and approximately 1,100 species distributed worldwide across tropical, subtropical, and warm temperate regions and inhabits a variety of marine, brackish water, and freshwater habitats (Strong et al. 2011). Despite their ubiquitousness in aquatic habitats, only three genome assemblies were available in the INSD database, and two of them are of low genome coverage with less than 25% of BUSCO completeness (supplementary table S1, Supplementary Material online). Our genome assembly is the first chromosome-scale assembly in this large molluskan superfamily.

We used about 250 Gb PacBio long-read sequences, 240 Gb Illumina paired short-read sequences, and 160 Gb Hi-C library sequences to assemble the genome of *S. habei* (supplementary table S2, Supplementary Material online). We estimated the genome size of *S. habei* as 1.95 Gb based on the *k*-mer distribution and 1.89 Gb based on the back-mapping technique. The genome size of the final assembly was about 1.98 Gb, slightly larger than the estimates based on the short-read sequences. Repeated elements often affect short-read sequence-based estimations, resulting in smaller genome sizes (Heckenhauer et al. 2022; Pfenninger et al. 2022). This perhaps explains the discrepancy between the assembly size and genome size estimations.

The final assembly is composed of 7,743 contigs and 578 scaffolds (table 1), and approximately 98.9% of sequences were contained in the eight megascaffolds. The mapping rate of the Illumina short reads against the final assembly was 99.7%, and 99.5% of the reads were paired. We estimated the total repeat content of the *S. habei* genome was about 76% (supplementary table S3, Supplementary Material online), which is similar to other freshwater and terrestrial snails with large genome sizes (∼3 Gb genome size, repeat content: 71–77%; Schell et al. 2017; Guo et al. 2019; Saenko et al. 2021) but is much larger compared with the genome sequences of other freshwater snails (genome size <1 Gb, repeat content: 11–48%; Gomes-dos-Santos et al. 2019). Long interspersed nuclear elements (LINEs) dominated about 20% of the genome (supplementary table S3, Supplementary Material online). Long terminal repeat (LTR) retrotransposon was the second largest group, occupying about 10% of the genome. Nearly 30% of the repeat elements were not classified into known categories.

**Table 1:** The Statistics for the Genome Assembly and Annotation for *S. habei*

Item	Category	Value
Assembly statistics	Assembled genome size	1,984,187,800
	Number of scaffolds	578
	Number of contigs	7,743
	N50	236,511,961
	N90	178,179,616
	GC content (%)	45.0
	BUSCO completeness (%)	94.4
	Single copy (%)	93.1
	Duplicated (%)	1.3
	Fragmented (%)	3.1
	Missing (%)	2.5
Protein-coding genes	Number of genes	41,547
	Number of transcripts	45,269
	Number of annotated transcripts	32,409
	Uniprot_Swiss-prot	14,367
	Uniprot_TrEMBL	28,904
	RefSeq (invertebrates)	28,789
	InterPro	30,114
	EggNOG	29,214
	With GO terms	24,676
	With KEGG pathways	8,983
	BUSCO completeness (%)	92.2
	Single copy (%)	89.0
	Duplicated (%)	3.2
	Fragmented (%)	4.0
	Missing (%)	3.8

The BUSCO completeness was estimated using the metazoan core gene database.

We annotated the genome by ab initio gene prediction based on 11 Gb of *S. habei* transcriptome sequences (supplementary table S2, Supplementary Material online). The final assembly contains 41,547 protein-coding genes (45,269 transcripts; table 1). The number of genes modeled in our assembly is larger than that of other freshwater snail genomes (14,000–24,000 genes), while this is comparable to that of some marine and terrestrial snails (Masonbrink et al. 2019; Saenko et al. 2021; Patra et al. 2023). The mean coding sequence length is 1,400 bp, and about 82% of genes have multiple exons (6.9 exons on average). Of the protein-coding genes modeled in our assembly, 32,409 genes were successfully annotated in one or more of the following databases: SwissProt, TrEMBL, EggNOG, InterPro, and RefSeq Invertebrate databases (table 1), and 24,676 genes possess gene ontology (GO) terms in the EggNOG database. The BUSCO analyses detected 94.4% of the metazoan core genes in the genome assembly and 92.2% in the annotated genes (table 1), suggesting the assembly covers the majority of genes present in the *S. habei* genome sequence. The result of the BlobTools analysis demonstrated the contamination from bacteria was negligible (supplementary fig. S1, Supplementary Material online).

Burch (1968) inferred that the basic haploid chromosome number of *S. habei* is seven (2*N* = 14) based on his karyotypic observations. He further reported three to six supernumerary chromosomes (B chromosomes) with variable chromosome lengths in *S. habei*. The sizes of these supernumerary chromosomes were comparable to those of the other chromosomes in *S. habei*. The Hi-C contact map demonstrates that there are seven well-resolved scaffolds and one scaffold with a complicated mosaic pattern (chr4 in fig. 1*B*). We consider that the seven well-resolved scaffolds correspond to the seven basic chromosomes. Because the supernumerary chromosomes often experience large structural rearrangements (Endo et al. 2008), we postulate that one scaffold with a mosaic pattern represents the complex of multiple supernumerary chromosomes. We ensured that the observed mosaicism was not the artifact of the long-read–based scaffolding since we got a similar mosaic pattern without the long-read–based scaffolding process. The supernumerary chromosomes are often not functional and contain no essential genes (Camacho et al. 2000). However, in our assembly, the amount and distribution pattern of coding genes and repeat elements are comparable to the other chromosomes (fig. 1*C*), suggesting chr4 is functional and perhaps essential for the survival of *S. habei*. Future genomic studies in combination with cytological techniques are required to evaluate the supernumerary status and the detailed genomic function of chr4.

Despite the large and repetitive nature of the *S. habei* genome, the assembly reported here is of high quality and will be an essential genomic resource for the evolutionary studies on the cerithioidean snails. In particular, it will substantially contribute to understanding the genomic background of the adaptive radiation of *Semisulcospira* in the ancient Lake Biwa.

## Materials and Methods

### Study Sample, DNA Extraction, and Quality Evaluation

We collected a male *S. habei* from Uji River, the main outlet of Lake Biwa. *Semisulcospira habei* is characterized by grid-like nodes on the shell surface and the elongated conical shell outlines with fewer number of basal cords on the body whorl (Davis 1969). High-molecular-weight genomic DNA (HMW-gDNA) was extracted from fresh snail tissue using cetyltrimethylammonium bromide (CTAB) and NucleoBond HMW DNA (Macherey-Nagel, Germany). In brief, about 110 mg of foot tissue was cut into small pieces (<0.5 mm^2^) and digested in 3 ml of 2× CTAB solution with 200 µl of Proteinase K solution at 50 °C for about 2 h. HMW-gDNA was washed once with 3 ml of chloroform, and the water phase containing HMW-gDNA was transferred to the NucleoBond HWM DNA column and cleaned up following manufacturer's protocol. In the final process of DNA extraction, we added 3.5 ml of isopropanol into 5 ml of Buffer H5 to precipitate DNA. The HMW-gDNA was briefly washed with 70% ethanol, air-dried for approximately 10 min, and dissolved in Tris-HCl buffer. The purity and concentration of the extracted HMW-gDNA were evaluated using BioSpec-nano spectrometer (Shimadzu, Japan) and Qubit fluorometer (Thermo Fisher Scientific, United States). The approximate length of the extracted DNA fragments was evaluated using a pulse-field agarose gel electrophoresis.

### Whole Genome Sequencing

We sequenced the genome of *S. habei* using two runs of the PacBio Sequel II sequencer. The PacBio library preparation for the continuous long reads (CLRs) and sequencing were performed with the SMRTbell Express Template Prep Kit 2.0 and the Sequel II Binding Kit 2.0/Sequencing Kit 2.0 at the National Institute of Genetics Japan (NIG). We also obtained the short-read sequences using the Illumina NovaSeq 6000 sequencer. The 150 bp paired-end sequencing was performed using the TruSeq DNA PCR-Free Library Prep Kit and the NovaSeq 6000 SP Reagent Kit v1.5 at NIG. We conducted the quality filtering of the short-read sequences using fastp (Chen et al. 2018) by eliminating the low-quality bases with a quality score less than Q30.

### Estimation of Genome Size

We used two short-read sequence-based approaches to estimate the genome size of *S. habei*. First, we estimated the genome size using the *k*-mer distribution. We used KMC (Kokot et al. 2017) to count canonical 21-mers from the short-read sequences and produced *k*-mer count histogram with a max coverage threshold of 1 million reads. The obtained histogram was analyzed by GenomeScope (Vurture et al. 2017) to estimate the genome size and average heterozygosity. Second, we used the back-mapping method, which estimates the genome size by dividing the total sum of the short-read sequences by the peak coverage from mapping back the final assembly (Schell et al. 2017). We used a Perl wrapper script (backmap.pl) of ModEst (Pfenninger et al. 2022) to execute the back-mapping process.

### De Novo Genome Assembly

The genome assembly of *S. habei* was constructed based on the PacBio long-read data set using Flye v. 2.9 (Kolmogorov et al. 2019) with genome size set to 2 Gb and assembly coverage of 100. Duplicated contigs were removed from the assembly using the long-read sequences by Purge_haplotigs v. 1.1.2 (Roach et al. 2018) and Redundans v. 0.14a (Pryszcz and Gabaldón 2016). The resultant assembly was used as input for further scaffolding using the long-read sequences by Longstitch v. 1.0.4 (Coombe et al. 2021). The assembly was then polished using the short-read sequences by Pilon v. 1.23. Finally, the paired-end RNA-seq data set was used for scaffolding the assembly using P_RNA_scaffolder (Zhu et al. 2018).

### Hi-C Scaffolding

We obtained the additional sequences using the Hi-C technology to achieve the chromosomal-scale assembly. To construct the Hi-C library, about 20 mg of columellar muscle tissue from the same individual was used for the genome sequencing. The Hi-C library was made using an Arima-HiC+ kit (Arima Genomics, United States) and sequenced using NovaSeq 6000. The library construction and sequencing were performed at NIG. The Hi-C data set was processed using Juicer (Durand et al. 2016b) followed by 3D-DNA v. 180114 (Dudchenko et al. 2017) to scaffold the genome assembly at the chromosomal scale. Juicebox v. 1.11.08 (Durand et al. 2016a) was used to manually review the assembly errors. We eliminated the scaffolds with more than 50% of ambiguous bases (or assembly gaps) and without any protein-coding genes, and the resulting 578 scaffolds were defined as the final assembly.

### Genome Annotation

We used RepeatModeler v. 2.0.3 (Flynn et al. 2020) to construct a species-specific library of transposable elements and repeats for *S. habei*. The obtained species-specific model was combined with the known repeat library of Mollusca from the RepeatMasker database. This combined library was used to detect and soft masked the repeat elements using RepeatMasker v. 4.1.4 (http://www.repeatmasker.org).

We performed the transcriptome sequencing using the tissues from the same individual to make the transcriptome database for *S. habei* (table 1). The head and middle part, including foot and mantle tissues, and the other part, including gonadal and hepatopancreas tissues, were fixed separately using RNAlater (Qiagen, United States). These two parts were used for the transcriptome library constructions and sequencing. The transcriptome sequencing was performed at Macrogen Japan. The RNA-seq data set was aligned to the final assembly using Hisat2 v. 2.2.1 (Kim et al. 2019). We used Braker2 v. 2.1.6 (Brůna et al. 2021) to achieve ab initio gene prediction with the aid of Augustus (Stanke et al. 2008) and GeneMark (Brůna et al. 2020). Untranslated regions were also detected using Gushr (Hoff and Stanke 2019). Protein sequences of the predicted genes were constructed using Gffread v. 0.12.7 (Pertea and Pertea 2020). We then performed functional annotations of the predicted genes using the EnTAP v. 0.10.8 pipeline (Hart et al. 2020) based on SwissProt, TrEMBL, EggNOG, InterPro, and RefSeq Invertebrate databases. We evaluated gene content completeness of the final assembly and annotation using BUSCO v.5 by counting the presence of the metazoan core genes (Manni et al. 2021). Finally, BlobTools v.1.1.1 (Laetsch and Blaxter 2017) was used to assess potential bacterial contaminations based on the NCBI nonredundant nucleotide database and the UniProt reference proteome database.

## Supplementary Material

evad208_Supplementary_Data

## Data Availability

The whole genome sequences of *S. habei* were deposited to DDBJ BioProject accession number PRJDB16287. The accession number of the PacBio long-read sequences is DRR494364, and that of the Illumina short-read sequences is DRR494361. The sequences of the Hi-C library were deposited at DDBJ under accession number DRR494363. The transcriptome short-read sequences were deposited under accession number DRR494362. The final chromosome-scale assembly was deposited at DDBJ under accession number BTPG01000001-BTPG01000578. The genome and genome annotation results have also been deposited in Figshare under the DOI: 10.6084/m9.figshare.24180666.
