# Supplementary material for: Chromosome-Scale Genome Assembly of the Freshwater Snail Semisulcospira habei from the Lake Biwa Drainage System
Source: Genome Biol Evol. 2023 Nov 28;15(11):evad208. doi: 10.1093/gbe/evad208 (PMC10683039; doi:10.1093/gbe/evad208)

Figure S1. The blobplot of *Semisulcospira habei* genome assembly. The scaffolds are represented by circles, and the colors indicate the taxonomic groups. The circles were plotted according to the GC proportion and read coverage. The rectangle panels exhibit the distribution of the total span (kb) of contigs for a given coverage (right panel) and for a GC proportion (upper panel).

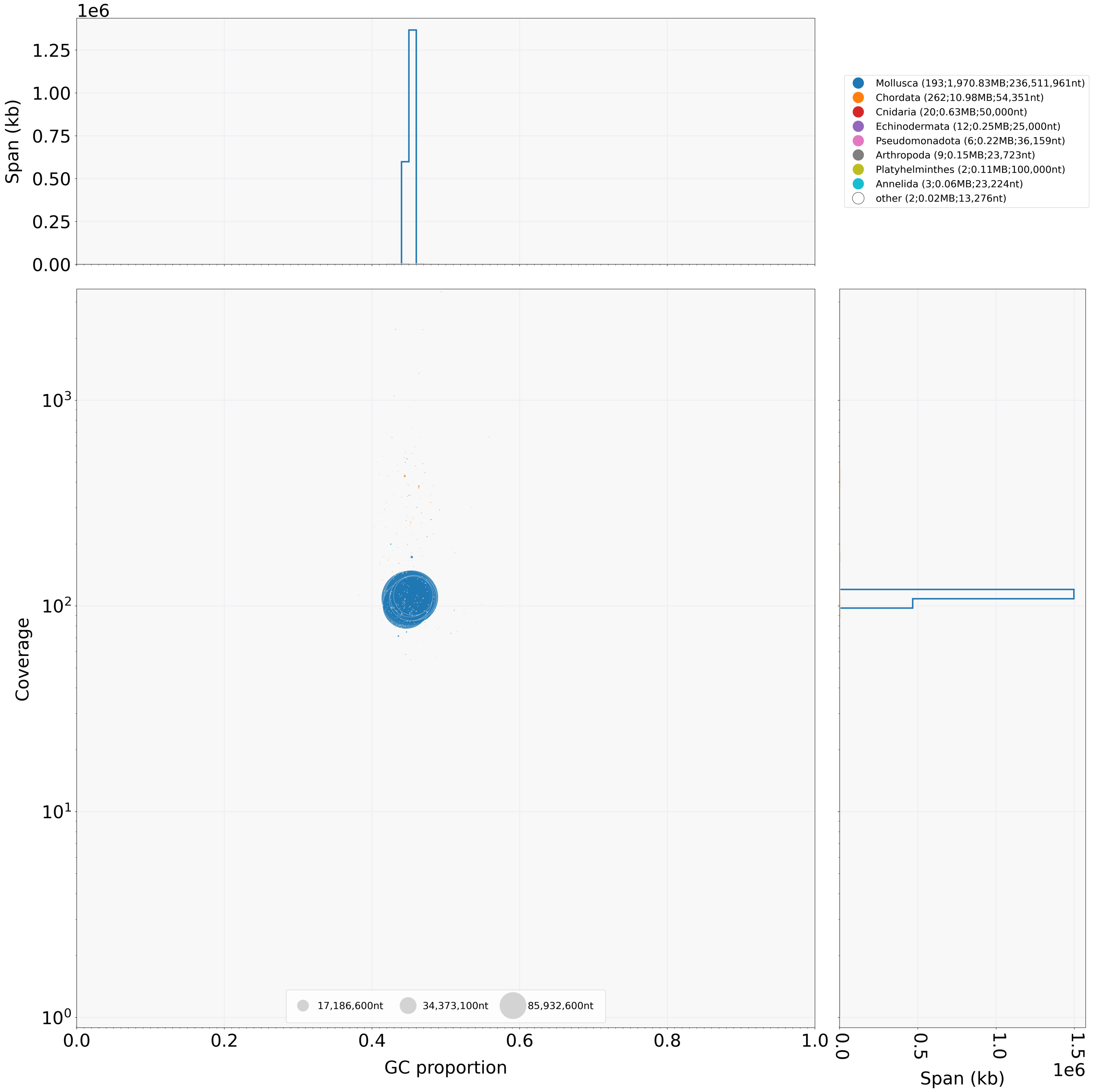

Supplement: evad208_Supplementary_Data [file evad208_supplementary_data.zip › FigureS1.pdf]
